# Late Onset Graft Plasmacytoma-Like PTLD Presenting as Acute Hyperglycemia in a Kidney-Pancreas Transplant Recipient

**DOI:** 10.1155/2019/2818074

**Published:** 2019-05-19

**Authors:** P. Ventura-Aguiar, M. T. Cibeira, A. Martinez, M. Cuatrecasas, M. Aymerich, J. Ferrer, J. Blade, F. Diekmann, M. J. Ricart

**Affiliations:** ^1^Department of Nephrology and Renal Transplant, Hospital Clínic, Barcelona, Spain; ^2^Department of Hematology, Hospital Clínic, Barcelona, Spain; ^3^Department of Pathology, Hospital Clínic, Barcelona, Spain; ^4^Department of Hepatobiliopancreatic Surgery, Hospital Clínic, Barcelona, Spain

## Abstract

Allograft infiltration has been described in up to 20% of all patients with posttransplant lymphoproliferative disorder (PTLD), most representing EBV-positive B-cell lymphomas. Plasma cells are often observed in humoral rejection biopsies, but graft infiltration by plasmacytoma-like PTLD is rare. We report the case of a 54-year-old simultaneous pancreas-kidney transplant recipient (immunosuppression: OKT3, methylprednisolone, cyclosporine, and azathioprine), diagnosed with an IgG-kappa monoclonal gammopathy of undetermined significance eighteen years after transplant. Nine months later, pancreas allograft biopsy performed due to new-onset hyperglycemia (HgA1C 8.6%, C-peptide 6.15ng/mL and anti-GAD 0.9UI/mL) revealed a monotypic plasma cell infiltrate, CD19, CD79a, CD138 positive, with IgG-kappa light chain restriction, and EBV negative. PET-scan FDG uptake was limited to pancreas allograft. Tumor origin could not be established (using DNA microsatellite analysis). Despite treatment with bortezomib and dexamethasone, patient eventually died one month later. This is the first report of a late onset extramedullary plasmacytoma involving a pancreas allograft.

## 1. Introduction

Posttransplant lymphoproliferative disorder (PTLD) is a frequent complication among solid organ transplant (SOT) recipients, with an incidence up to 5.72% [1]. Several factors have been associated with PTLD risk, such as induction therapy, recipient age, HLA mismatching, transplant era, or type of transplanted organ [1].

The vast majority of PTLDs' corresponds to EBV-associated monotypic B-cell lymphomas, with graft involvement in up to 20% of all cases [2].

Polyclonal allograft infiltrating plasma cells have been described during rejection episodes [3]. Continuous antigen exposure leading to localized lymphoid proliferation of donor-specific antibody-producing plasma cells [4] may promote a switch towards monoclonal immunoglobulin production and finally overtly malignant monomorphic plasmacytoma-like graft infiltration [5].

Herein we report the case of an extramedullary plasmacytoma-like PTLD localized to the pancreas allograft in a recipient of a simultaneous kidney-pancreas transplant diagnosed on a biopsy performed due to new-onset hyperglycemia.

## 2. Case Report

A 54-year-old male with a history of type 1 diabetes mellitus and biopsy confirmed IgA nephropathy and received a simultaneous pancreas-kidney (SPK) transplant in 1996. He received induction immunosuppression with OKT3, methylprednisolone (MPN), cyclosporine A (CyA), and azathioprine (AZA). The immediate posttransplant period was complicated with acute cellular grade Ia kidney rejection (treated with three bolus of MPN), and a biopsy confirmed Herpes simplex virus (HSV) and cytomegalovirus (CMV) esophagitis (treated with acyclovir and ganciclovir, respectively). At six months, seroconversion of hepatitis C virus (HCV) was diagnosed following transient elevation of transaminases and cholestasis. HCV infection route could not be determined, since donor was HCV negative (ELISA), and HCV detection (ELISA) was standard protocol at hospital blood bank previous to blood transfusions. Azathioprine was withdrawn due to the hepatotoxicity risk. Follow-up from month 6 onwards was uneventful while on maintenance immunosuppression with CSA and prednisone 5mg and with functioning allografts (serum creatinine 0.9mg/dL, HbA1c <6% and normal serum amylase and lipase). Despite positive HCV viremia (latest RNA determination with 1.637.000 copies/mL), a conservative management was decided due to the increased treatment induced rejection risk.

In October 2014, patient was diagnosed with an IgG-kappa monoclonal gammopathy of undetermined significance (serum M protein of 24 g/L, serum *κ*/*λ* ratio of 5.2, proteinuria of 249 mg/24h with the presence of monoclonal IgG-kappa by immunofixation in urine, and <10% plasma cells in the bone marrow aspirate), without end-organ damage that could be attributed to the gammopathy. Both grafts presented normal function (HgA1C 5.9%, serum creatinine 1.5mg/dL).

**Figure 1:**
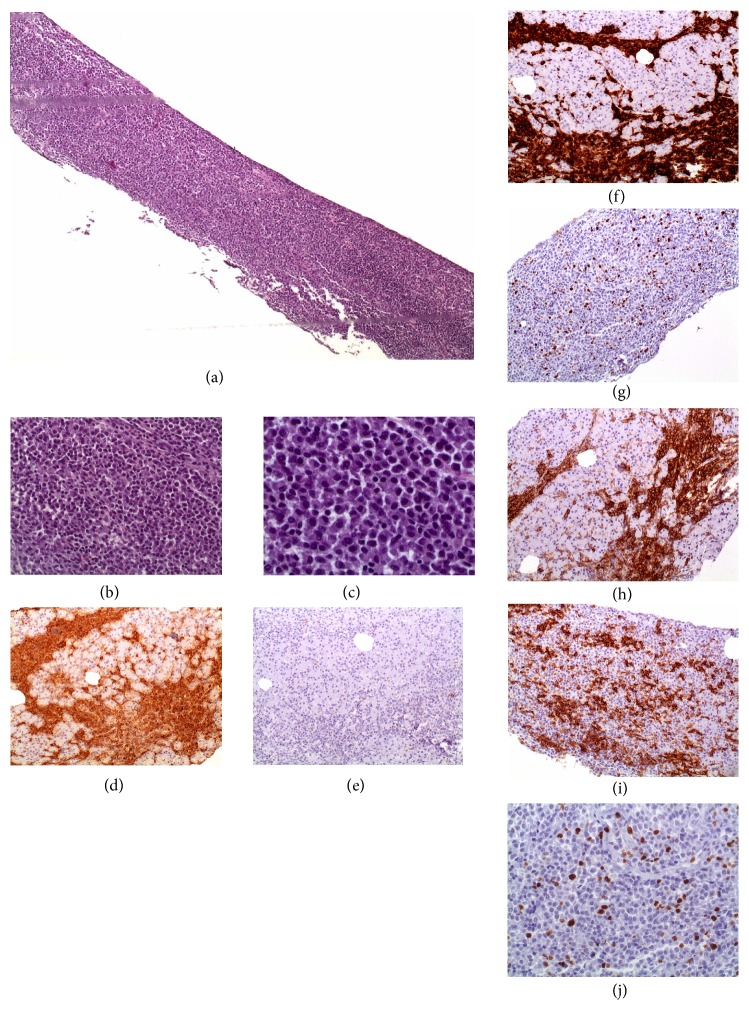
*Plasma cells infiltration of pancreas allograft on indication biopsy due to elevation of amylase and lipase*. (a–c) Extensive diffuse infiltration and pancreatic tissue replacement with plasma cells observed on H&E ((a): X40; (b): x200; (c): x400). (d) Extensive immunostaining for IgG-kappa chains, x100; (e) absence of lambda staining, x100; (f) immunohistochemical staining were positive for CD79 plasma cells, x100; (g) negative for CD20, x100; and (h) extensively positive for CD138, x100; (i) and (j) Ki67 proliferation index was under 3%.

**Figure 2:**
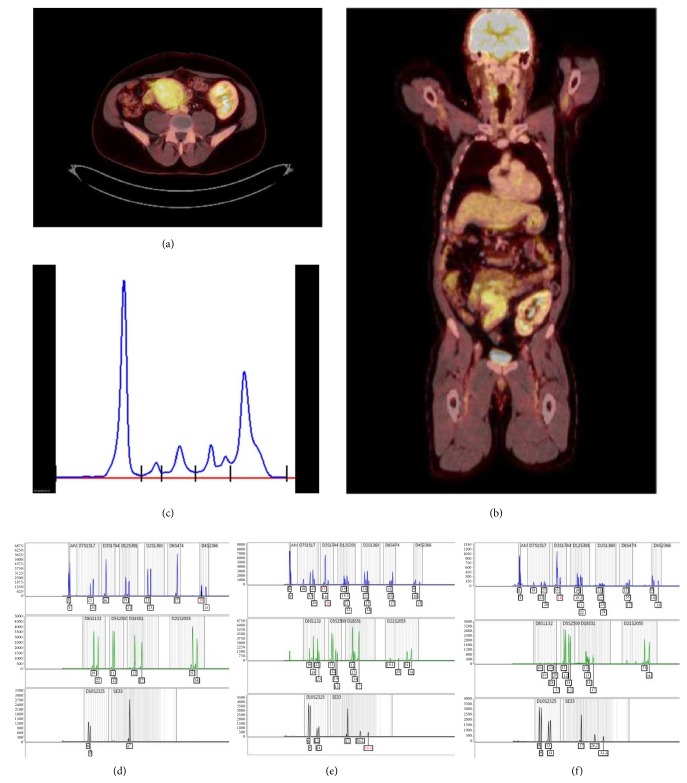
*PET/CT scan (a) and (b)*: diffuse uptake of FDG and enlargement of pancreas allograft (arrow), without focal lesions. Normal captions of bone marrow, brain, and kidney (arrow heads); (c)* serum proteinogram*: typical M protein gamma peak; (d)* microsatellite analysis* (Mentype® Chimera®): DNA from recipient peripheral blood (leukocytes). (e-f) Mixed donor and recipient microsatellite analysis was observed in the DNA extracted from the paraffin block obtained from selected areas with a high infiltration of plasma cells.

Nine months later (19 years after transplant) patient presents a new-onset hyperglycemia (HgA1C 8.6%, C-peptide 6.15ng/mL, anti-GAD 0.9U/mL). Ultrasound revealed a globular and heterogeneous pancreas allograft, with biopsy evidencing a diffuse monotypic plasma cell infiltrate, with scarce normal parenchyma observed. Plasma cells were CD19, CD79a, CD138, and IgG-*κ* positive, with a cell proliferation index (Ki-67) under 3% (Figure 1). In situ hybridization for EBV (EBER) was negative. Serum calcium and hemoglobin remained normal, and kidney function was stable (serum creatinine 1.4mg/dL). Serum M protein had increased up to 34 g/L (Figure 2(c)), serum *κ*/*λ* ratio was 8.2, urinary protein excretion was normal, and 7% plasma cells were with abnormal phenotype identified on a bone marrow aspirate. Solid-phase Luminex® bead array was negative for HLA class I and class II antibodies. Positron emission tomography (PET) scan revealed a fludeoxyglucose (FDG) uptake limited to pancreas allograft (Figures 2(a) and 2(b)). Serum EBV and HHV-6 PCR were negative.

Tumor origin determination was attempted using DNA extracted from a highly infiltrated section of the paraffin block. HLA phenotyping revealed a mixed chimerism (Figures 2(d)–2(f)). Sex mismatching was unsuitable for determination of tumor origin (male donor).

Diagnosis of extramedullary plasmacytoma localized to the pancreas allograft was established and treatment with bortezomib and dexamethasone initiated, following CSA withdrawal. Patient eventually died one month following the beginning of treatment due to spontaneous cerebral hemorrhage, secondary to a hypertensive emergency.

## 3. Discussion

Extramedullary plasmacytoma is a rare disease, representing less than 5% of all plasma cell dyscrasias [6]. Contrary to mixed cryoglobulinemia, in which a correlation is well established, no clear association between hepatitis C and multiple myeloma has been established to date [7]. Despite the absence of a known cause-effect of hepatitis C in plasma cell dyscrasias, several cases of extramedullary plasmacytoma in patients with hepatitis C infection, such as the one herein presented, have been reported in the literature [6, 8–11].

B- and T-cell lymphomas are a common form of PTLD, most of which are EBV-related [2]. Plasma cell dyscrasias are a rare form of PTLD [2], with extramedullar plasmacytoma accounting for less than 4% of all cases [12]. A late onset, as in the case herein reported (19 years after transplant), is frequent. In two retrospective analysis [5, 12], plasmacytoma was diagnosed more than 10 years after SOT in up to 59% of cases. These studies also highlight the variability between clinical presentation, treatment options, and outcomes.

Allograft involvement as a manifestation of plasmacytoma-like PTLD has been previously described in SOT, infiltrating liver [5, 12], kidney [13], and heart [14] transplants. Pancreas graft infiltration by an EBV-positive B-cell lymphoma was described by Rehbinder et al. [15]. To our knowledge, this is the first report of an isolated pancreas allograft plasmacytoma-like PTLD.

A common feature of allograft restricted plasmacytoma-like involvement is the absence of the systemic features of plasma cell dyscrasias. Lytic bone lesions and elevated serum calcium are often absent, and the bone marrow plasma cells infiltration is low [5, 12]. Therefore, in the presence of monoclonal gammopathy of undetermined significance and allograft dysfunction, a plasmacytoma-like diagnosis should be suspected. The case herein presented suggests a potential role of the PET-scan to increase diagnostic accuracy in those cases where biopsy cannot be performed [13, 16].

The pathophysiology of plasmacytoma-like PTLD with isolated graft involvement is yet to be established. Chronic antigen exposure and consequent B-cell stimulus in the germinal center of lymph nodes, leading to an immunoglobulin switch and clonal expansion, may explain allograft restricted pathology [5]. Two premises support this hypothesis.

First, it has been suggested that chronic antigen exposure increases the risk of plasma cell dyscrasias. In Gaucher's disease, Nair et al. [17] identified an association between lyso-glucosylceramide (LGL1) continuous antigen exposure and the increased risk of multiple myeloma in this population. In a SOT setting, a similar pathogenesis is likely to exist, with the frequent late onset of the disease supporting this hypothesis [5, 18]. Moreover, allograft plasma cell infiltration is a well-documented histopathology finding and has been associated with antibody-mediated rejection (and worse response to treatment) [3]. The importance of local allograft lymphoid tissue in the development of immune responses has been recently highlighted in heart transplant [19].

Multiple myeloma is a clonal B-cell neoplasm of fully differentiated B cells. It has been proposed that, following the switch of immunoglobulin class, plasma cells migrate to bone marrow, where they find the microenvironment that enables them to mature and expand [20]. Hubier et al. have recently identified mature donor-specific antibody-producing plasma cells localized to allograft ectopic lymphoid structures [4].

In our case, single antigen assay was not performed, but solid-phase single bead Luminex® screening was negative the day of pancreas biopsy, being therefore unlikely to present a donor-specific antibody (DSA). Recently, Hamada et al. described a case series of kidney transplant recipients with plasma cell rich acute rejection (PCAR) [21]. Of the 6 patients, only two were characterized as presenting an acute rejection, and all the remaining four cases did not present DSAs. In the setting of immunosuppression, these could undergo a local clonal expansion without the need of the bone marrow microenvironment. Molecular analysis to graft biopsies has been expanding the understanding of the interplay between immune cells in SOT. The molecular microscope has been exposing some yet unknown mechanisms occurring during episodes of acute rejection. Through a transcriptomic analysis, expression of molecules associated with antibody-mediated rejection (AMR) in the absence of DSAs and/or incomplete histopathological features of AMR is thought to increase diagnosis accuracy [22–24]. These results highlight the fact that antigen exposure leading localized plasma cell stimulation may be present despite the absence of DSAs.

Tumor origin in PTLD is often difficult to establish. Olagne et al. [25] identified tumor origin of 43 cases using a variety of analysis (FISH hybridization for sex mismatched cases, HLA immunostaining, and microsatellite analysis). Similarly, we have performed a microsatellite analysis using DNA extracted from an area with >80% of plasma cell infiltration (as previously described [25]) but were still unable to determine tumor origin due to the presence of a mixed chimerism. FISH hybridization was unsuitable for this case (no sex mismatching). Since patient autopsy was performed in another hospital, microsatellite analysis could only be performed using DNA extracted from the pancreas graft biopsy paraffin block. Nevertheless, the observed mixed chimerism suggests a recipient tumor origin, donor DNA being representative of pancreas acinar tissue and recipient from plasma cells. Shall the tumor have been donor-derived, recipient DNA expression in such a highly infiltrated area would be expected to be marginal (or absent). Additionally, and based on the French analysis [25] in which PTLD of donor origin presented significantly earlier than those of recipient origin (64% versus 22% <12 months after transplant, average time to PTLD 20 ± 28 versus 69 ± 68 months, respectively), the 228 months elapsed from pancreas transplant to disease diagnosis support the hypothesis of recipient origin.

Treatment of extramedullary plasmacytomas is not consensual and several different approaches have been reported, including surgery, chemotherapy, radiation, or surveillance [16]. PTLD allograft infiltration has been described in up to 20% of all cases and is usually associated with a better prognosis [1, 18]. In the setting of graft infiltrating plasmacytoma, surgical approach with transplantectomy, with or without adjuvant chemotherapy, may be attempted as a curative approach. The psychological burden it represents (patient return to dialysis or to insulin therapy if kidney or pancreas graft affected, respectively) is not to be underrated. Chemotherapy with protocols similar to those used to multiple myeloma, with melphalan, bortezomib, thalidomide, and plus steroids, have been described with conflicting outcomes [16]. As proposed by Caillard et al., an early diagnosis due to organ dysfunction prompts a timely institution of chemotherapy, immunosuppression withdrawal, and even transplantectomy, leading to better long-term outcomes [5, 18]. In the case herein reported, treatment with steroids and bortezomib was attempted, but patient premature death precludes conclusions regarding its efficiency.

In conclusion, we report the first case of an extramedullary plasmacytoma involving a pancreas allograft, presenting as new-onset hyperglycemia 19 years after transplant. Tumor origin could not be established, but long transplant vintage and current evidence for plasmacytoma pathophysiology suggest a recipient origin.

## Abbreviations

Anti-GAD: Glutamic Acid Decarboxylase Autoantibodies
AZA: Azathioprine
CMV: Cytomegalovirus
CSA: Cyclosporine A
EBV: Epstein-Barr Virus
ELISA: Enzyme-linked immunosorbent assay
FDG: Fludeoxyglucose
HCV: Hepatitis C Virus
HHV-6: Human Herpesvirus 6
HSV: Herpes Simplex Virus
LGL1: Lyso-glucosylceramide
MPN: Methylprednisolone
PET: Positron emission tomography
PTLD: Posttransplant lymphoproliferative disorder
SOT: Solid organ transplant
SPK: Simultaneous pancreas-kidney.

## References

[1] Caillard S., Lamy F. X., Quelen C. (2012). Epidemiology of posttransplant lymphoproliferative disorders in adult kidney and kidney pancreas recipients: report of the French registry and analysis of subgroups of lymphomas. *American Journal of Transplantation*.

[2] Caillard S., Lelong C., Pessione F., Moulin B. (2006). Post-transplant lymphoproliferative disorders occurring after renal transplantation in adults: report of 230 cases from the French registry. *American Journal of Transplantation*.

[3] Alexander J., Chu W., Swanson P. E., Yeh M. M. (2012). The significance of plasma cell infiltrate in acute cellular rejection of liver allografts. *Human Pathology*.

[4] Huibers M. M. H., Gareau A. J., Beerthuijzen J. M. T. (2017). Donor-specific antibodies are produced locally in ectopic lymphoid structures in cardiac allografts. *American Journal of Transplantation*.

[5] Karuturi M., Shah N., Frank D. (2013). Plasmacytic post-transplant lymphoproliferative disorder: a case series of nine patients. *Transplant International*.

[6] Cormio L., Mancini V., Calò B., Selvaggio O., Mazzilli T., Sanguedolce F. (2017). Asymptomatic solitary bladder plasmocytoma. *Medicine (Baltimore)*.

[7] Zignego A. L., Giannini C., Ferri C. (2007). Hepatitis C virus-related lymphoproliferative disorders: an overview. *World Journal of Gastroenterology*.

[8] Bigot-Corbel E., Gassin M., Corre I., Carrer D. L., Delaroche O., Hermouet S. (2008). Hepatitis C virus (HCV) infection, monoclonal immunoglobulin specific for HCV core protein, and plasma-cell malignancy. *Blood*.

[9] Di Micco P., Niglio A., Torella R., Di Micco B. (2002). Solitary plasmacytoma of the jaw occurring in an elderly woman affected by hepatitis C virus infection: a case report. *Tumori*.

[10] Ueda K., Matsui H., Watanabe T. (2010). Spontaneous rupture of liver plasmacytoma mimicking hepatocellular carcinoma. *Internal Medicine*.

[11] Brannan P. A., Cetinkaya A., Kim A. S., Palkovacs E. M. (2009). Solitary orbital plasmacytoma associated with chronic hepatitis C: a case report. *Orbit*.

[12] Trappe R., Zimmermann H., Fink S. (2011). Plasmacytoma-like post-transplant lymphoproliferative disorder, a rare subtype of monomorphic B-cell post-transplant lymphoproliferation, is associated with a favorable outcome in localized as well as in advanced disease: a prospective analysis of 8 cases. *Haematologica*.

[13] Kuppachi S., Naina H. V., Self S., Fenning R. (2013). Plasmacytoma-like post-transplantation lymphoproliferative disorder confined to the renal allograft: a case report. *Transplantation Proceedings*.

[14] Short S. R. P., Cook S. L., Kim A. S., Lamour J. M., Lowe E. J., Petersen W. C. (2016). Plasmacytoma-like posttransplant lymphoproliferative disorder in a pediatric heart transplant recipient. *Journal of Pediatric Hematology/Oncology*.

[15] Rehbinder B., Wullstein C., Bechstein W. O. (2006). Epstein-barr virus-associated posttransplant lymphoproliferative disorder of donor origin after simultaneous pancreas-kidney transplantation limited to pancreas allograft. *American Journal of Transplantation*.

[16] Pham A., Mahindra A. (2019). Solitary plasmacytoma: a review of diagnosis and management. *Current Hematologic Malignancy Reports*.

[17] Nair S., Branagan A. R., Liu J., Boddupalli C. S., Mistry P. K., Dhodapkar M. V. (2016). Clonal immunoglobulin against lysolipids in the origin of myeloma. *The New England Journal of Medicine*.

[18] Cibeira M. T., Lopez-Guillermo A., Colomer D. (2003). Diffuse large B-cell lymphoma arising from donor lymphoid cells after renal and pancreatic transplantation. *Annals of Hematology*.

[19] Huibers M. M. H., Gareau A. J., Vink A. (2015). The composition of ectopic lymphoid structures suggests involvement of a local immune response in cardiac allograft vasculopathy. *The Journal of Heart and Lung Transplantation*.

[20] Tricot G. (2000). New insights into role of microenvironment in multiple myeloma. *The Lancet*.

[21] Hamada A. M., Yamamoto I., Kawabe M. (2018). Clinicopathological features and outcomes of kidney allografts in plasma cell-rich acute rejection: a case series. *Nephrology*.

[22] Aubert O., Loupy A., Hidalgo L. (2017). Antibody-mediated rejection due to preexisting versus de Novo donor-specific antibodies in kidney allograft recipients. *Journal of the American Society of Nephrology*.

[23] Hidalgo L. G., Einecke G., Allanach K. (2008). The transcriptome of human cytotoxic T cells: MeasurIng the Burden of CTL-associated transcripts in human kidney transplants. *American Journal of Transplantation*.

[24] Hidalgo L. G., Sis B., Sellares J. (2010). NK cell transcripts and NK cells in kidney biopsies from patients with donor-specific antibodies: dvidence for NK cell involvement in antibody-mediated rejection. *American Journal of Transplantation*.

[25] Olagne J., Caillard S., Gaub M. P., Chenard M. P., Moulin B. (2011). Post-transplant lymphoproliferative disorders: Determination of donor/recipient origin in a large cohort of kidney recipients. *American Journal of Transplantation*.

